# Disrupted Myelination in FAHN: Insights from a Patient-Specific hiPSC Neuron–Oligodendrocyte Model

**DOI:** 10.3390/cells14161261

**Published:** 2025-08-15

**Authors:** Fatima Efendic, Andreas Hermann, Moritz J. Frech

**Affiliations:** 1 Translational Neurodegeneration Section “Albrecht Kossel”, Department of Neurology, University Medical Center Rostock, 18147 Rostock, Germany; 2 Center for Transdisciplinary Neurosciences Rostock (CTNR), University Medical Center Rostock, 18147 Rostock, Germany; 3 German Center for Neurodegenerative Diseases (DZNE), Rostock/Greifswald, 18147 Rostock, Germany

**Keywords:** FAHN, FA2H, oligodendrocytes, neurons, demyelination, autophagy, myelin proteins, induced pluripotent stem cells

## Abstract

Fatty-acid-hydroxylase-associated neurodegeneration (FAHN) is a rare neurodegenerative disorder caused by loss-of-function mutations in the *FA2H* gene, leading to impaired enzymatic activity and resulting in myelin sheath instability, demyelination, and axonal degeneration. In this study, we established a human in vitro model using neurons and oligodendrocytes derived from induced pluripotent stem cells (hiPSCs) of a FAHN patient. This coculture system enabled the investigation of myelination processes and myelin integrity in a disease-relevant context. Analyses using immunofluorescence and Western blot revealed impaired expression and localisation of key myelin proteins in oligodendrocytes and cocultures. FA2H-deficient cells showed reduced myelination, shortened internodes, and disrupted formation of the nodes of Ranvier. Additionally, we identified autophagy defects—a hallmark of many neurodegenerative diseases—including reduced p62 expression, elevated LC3B levels, and impaired fusion of autophagosomes with lysosomes. This study presents a robust hiPSC-based model to study FAHN, offering new insights into the molecular pathology of the disease. Our findings suggest that *FA2H* mutations compromise both the structural integrity of myelin and the efficiency of the autophagic machinery, highlighting potential targets for future therapeutic interventions.

## 1. Introduction

Fatty-acid-hydroxylase-associated neurodegeneration (FAHN) is a rare neurodegenerative disorder classified within the group of neurodegeneration with brain iron accumulation (NBIA) syndromes [1]. FAHN is caused by autosomal recessive mutations in the enzyme fatty acid 2-hydroxylase, encoded by the *FA2H* gene [2]. In addition to FAHN (OMIM #611026), mutations in *FA2H* have also been associated with leukodystrophy (OMIM #612319) [3,4] and hereditary spastic paraplegia (HSP) type SPG35 (OMIM #612319) [5,6]. To date, more than 100 disease-causing *FA2H* mutations have been reported in the Human Gene Mutation Database [7,8,9,10,11]. Approximately 90% of these are missense mutations located within the cytochrome b5 domain [3,6,12], which have been shown to reduce enzymatic activity [13]. FA2H is a three-domain lipid biosynthetic enzyme localised to the endoplasmic reticulum. It catalyses the synthesis of 2-hydroxy fatty acids, which are incorporated into ceramides and serve as precursors for galactosylceramides (GalCer)—major structural components of the myelin sheath [13,14]. Myelin is a lipid-rich membrane formed by oligodendrocytes (OLs) in the central nervous system and Schwann cells in the peripheral nervous system [14]. These glial cells ensheath axons, providing both metabolic support and increased conduction velocity [15,16]. Proper myelination depends on the compaction and biochemical stability of the myelin membrane, which is often compromised in neurodegenerative disorders [17], including FAHN.

Myelin membranes have a distinct lipid composition, comprising approximately 30% GalCer and sulfatide, of which nearly half contain 2-hydroxy fatty acids [13,14]. Given FA2H’s role in synthesising hydroxylated fatty acid GalCer (hFA-GalCer), FA2H deficiency is thought to cause abnormal myelin lipid composition, demyelination, myelin sheath instability, and subsequent axonal degeneration [14,18]. Mouse models with both conditional [2,19] and total *FA2H* knockout [20] have demonstrated that FA2H is not essential for initial myelin formation but is critical for long-term maintenance. This finding aligns with clinical observations in FAHN patients, who show initially normal development followed by progressive neurological decline—suggesting that the disease primarily results from defective maintenance rather than impaired formation of myelin [2,19,20]. In 18-month-old *FA2H*^−^/^−^ mice, reductions in myelin sheath thickness, instability, and disruption of compact myelin were observed [2,19]. While the exact sequence of pathological events remains unclear, available data suggest that these impairments may arise in a partially overlapping rather than strictly sequential manner and may mutually reinforce one another. The timing and extent of each process likely depend on the disease model and stage examined.

Beyond myelin-related changes, recent findings from a *Drosophila melanogaster FA2H*^−^/^−^ model and FAHN-patient-derived fibroblasts revealed additional impairments in mitochondrial dynamics and autophagic processes, suggesting a broader cellular pathology associated with FA2H dysfunction [21]. Nevertheless, our understanding of FAHN pathophysiology and the cellular consequences of FA2H deficiency remains limited, in part because current animal models do not fully replicate the human condition. This knowledge gap raises key questions about which alterations occur in a human model of FAHN and how changes in the lipid composition of myelin proteins contribute to axonal degeneration. To address this, we established a human-induced pluripotent stem cell (hiPSC) model and generated neural cells, including a coculture of oligodendrocytes and neurons.

Consequently, the aim of this study was to investigate disturbances in the expression of key myelin-structuring proteins, such as myelin basic protein (MBP) and proteolipid protein (PLP), and to examine how such alterations may disrupt the architecture of the nodes of Ranvier. These disruptions could contribute to the pathophysiological cellular phenotype of FAHN, particularly in the context of impaired myelin maintenance and axonal degeneration. To this end, we conducted immunocytochemical analyses to assess the expression and localisation of myelin-related proteins in both monocultures and cocultures. The structural integrity of the nodes of Ranvier was examined using confocal microscopy and specific marker proteins, enabling detailed evaluation of nodal organisation. In addition to analysing myelination-related changes, we also investigated the role of autophagy in FAHN, based on prior evidence linking FA2H dysfunction to impaired autophagy and mitochondrial abnormalities [21]. Given the established connections between lipid metabolism, ceramide accumulation, and autophagy, we aimed to determine whether autophagic dysfunction contributes to the cellular phenotype of FAHN. To this end, we utilised established autophagy markers and performed immunofluorescence and colocalisation analyses on neurons and oligodendrocytes. Additionally, Western blot experiments were conducted to assess FA2H expression, key myelin-structuring proteins, and core components of the autophagy pathway.

## 2. Materials and Methods

### 2.1. hiPSC Cultivation

The hiPSC line AKOSi006-A (hereafter referred to as CTRL) was employed as a control [22]. The cell line AKOSi010-A, derived from patient fibroblasts, carries the compound heterozygous mutation p.Gly45Arg/p.His319Arg (hereafter referred to as FAHN). The fibroblasts were obtained from Dr Sunita Venkateswaran at the Children’s Hospital of Eastern Ontario in Ontario, Canada. The generation and characterisation of AKOSi010-A hiPSCs from patient-specific fibroblasts were recently published [23]. The hiPSCs were cultured on Matrigel-coated (Corning, Corning, NY, USA) plates in mTeSR medium (STEMCELL Technologies, Cologne, Germany), with the medium renewed daily. The cells were passaged every five days and detached using ReLeSR (STEMCELL Technologies, Cologne, Germany). They were maintained at 37 °C and 5% CO_2_ in a humidified incubator. Following each passage, the mTeSR medium was supplemented with 10 μM Y-27632 (STEMCELL Technologies, Cologne, Germany).

### 2.2. Neural Differentiation

Once the hiPSCs had reached 70–80% confluency, the hiPSC medium was removed and the cells were washed with DMEM/F12. The medium was then replaced with 2 mL of neural induction medium (NIM), supplemented with 10 μM Y-27632 (STEMCELL Technologies, Cologne, Germany). Undifferentiated colonies were manually detached and transferred onto poly-L-ornithine (PLO, 15 μg/mL; Sigma-Aldrich, Taufkirchen, Germany) and laminin-coated (10 μg/mL; Trevigen, Gaithersburg, MD, USA) plates in NIM supplemented with 5 ng/mL basic fibroblast growth factor (FGF2), 20 μM SB-431542 (Tocris Bioscience, Wiesbaden, Germany), and 1 μM LDN193189 (Sigma-Aldrich, Taufkirchen, Germany) to induce the formation of neural rosettes. The medium was changed daily until day 9. On day 9, after neural rosettes had formed, NIM was replaced with neural progenitor cell (NPC) medium, supplemented with 10 ng/mL FGF2 and 10 ng/mL epidermal growth factor (EGF; PeproTech, Hamburg, Germany). On day 14, non-differentiated cells were manually removed. Neural rosettes were detached using Accutase, and neural progenitor cells (NPCs) were isolated using magnetic beads (Miltenyi Biotec, Teterow, Germany) targeting the surface marker polysialylated-neural cell adhesion molecule (PSA-NCAM), according to the manufacturer’s instructions. Purified NPCs were seeded onto PLO/laminin-coated plates in N2B27 medium composed of 40% neurobasal medium (Thermo Fisher Scientific, Dreieich, Germany), 60% DMEM/F12 (Fisher Scientific GmbH, Schwerte, Germany), 1 × B27, 1 × N2, and 0.5% penicillin/streptomycin, further supplemented with 10 ng/mL FGF2 and 10 ng/mL EGF. The NPC medium was renewed every three to four days, and cells were passaged with Accutase (Sigma-Aldrich, Taufkirchen, Germany) every five days. Neuronal differentiation of NPCs into a mixed population of TUJ1- and MAP2-positive neurons and GFAP-positive astrocytes occurred spontaneously. NPCs were seeded at an expansion density of 100,000 cells/cm^2^ on PLO/laminin-coated plates in proliferation medium (N2B27 without growth factors; Thermo Fisher Scientific, Dreieich, Germany). The proliferation medium was changed every four to six days over a six-week period.

### 2.3. Lentivirus Virus Generation

Viral vectors, proteins, and enzymes—including vesicular stomatitis virus glycoprotein G (VSV-G) and the packaging plasmid psPAX2—were used for lentivirus production. For this purpose, 3.0 × 10^6^ HEK293T cells were seeded into two 10 cm cell culture dishes containing basal medium without penicillin/streptomycin. The cells were cultured overnight at 37 °C, 5% CO_2_, and 90% relative humidity before transfection. In two separate 1.5 mL tubes, 180 μL of Opti-MEM Reduced Serum Medium (Thermo Fisher Scientific, Dreieich, Germany) was mixed with 20 μL of Lipofectamine™ 2000 (Thermo Fisher Scientific, Dreieich, Germany)

### 2.4. Generation of Oligodendrocyte Progenitor Cells

The protocol for the generation of oligodendrocyte progenitor cells (OPCs) was adapted from a recently published protocol [24]. The initial step involved the use of hiPSCs, which were passaged using Accutase supplemented with 10 μM Y-27632. Upon reaching 40–50% confluency, the cells were transferred to N2B27 medium containing small-molecule inhibitors of the SMAD pathway: 20 μM SB431542 (Tocris Bioscience, Wiesbaden, Germany) and 5 ng/mL LDN193189 (Miltenyi Biotec GmbH, Teterow, Germany), as well as 100 μM retinoic acid (RA; Sigma-Aldrich, Taufkirchen, Germany). On day 5, a sonic hedgehog agonist (also known as a smoothened agonist or SAG; Sigma-Aldrich, Taufkirchen, Germany) was added to the N2B27 medium already containing SB431542 and LDN193189. The addition of 10 μM SAG resulted in a significant proportion of OLIG2^+^ cells after eight days of neural induction. Subsequently, the cells were dissociated into single units using Accutase and seeded at a density of 50,000–75,000 cells/cm^2^ on PLO/laminin-coated plates in N2B27 medium supplemented with 10 μM Y-27632. After 24 h, the cells were transduced with lentivirus vectors encoding FUW-SOX10, FUW-M2rtTA, pMD2.G, and psPAX2. Viral titres were pre-titrated and calculated to achieve a multiplicity of infection (MOI) of 1.5. On the following day, the viral medium was replaced with oligodendrocyte (OL) differentiation medium supplemented with 10 ng/mL PDGF-AA (PeproTech, Hamburg, Germany), 10 ng/mL IGF-1, and 5 ng/mL HGF (both Sigma-Aldrich, Taufkirchen, Germany).

### 2.5. Flow Cytometery

To assess the purity of O4 expression by fluorescence-activated cell sorting (FACS), a small aliquot of cells (50,000–100,000 cells) was collected. To evaluate O4^+^ purity and determine total cell count (O4^+^ cells typically constitute ~10% of the starting population), the cell pellet was resuspended in 1 mL of OL induction medium supplemented with ROCK inhibitor. First, the cell aliquot was resuspended in 95 μL of ice-cold MACS buffer. Then, 5 μL of O4-APC antibody was added and incubated for 15 min at 4 °C. The cells were washed with 2 mL of MACS buffer, centrifuged, resuspended in 200 μL of MACS buffer, and subsequently analysed on a flow cytometer. Appropriate isotype or unstained control samples were used to define gating thresholds.

### 2.6. Coculture Systems

To study the myelination process, it was necessary to obtain mature neurons and mature oligodendrocytes (OLs) expressing myelin proteins. This was achieved by differentiating a coculture of neurons, astrocytes, and oligodendrocytes. Accordingly, differentiation of neuronal differentiated cells (NDCs) was initiated several weeks in advance. Once both cell types had been obtained, the NDC medium was replaced with coculture medium supplemented with 1 μg/mL doxycycline (applied only for the first four days), 1 × Y-27632, and 10 μg/mL laminin. Subsequently, OLs were seeded onto mature neurons at a density of 50,000 O4^+^ cells/cm^2^. The cells were incubated at 37 °C for four days, after which doxycycline was removed from the coculture medium. The cells were then cultured for a further 35–40 days, with partial medium changes two to three times per week. This was performed by gently replacing half of the medium with fresh medium. At the conclusion of the coculture period, the medium was discarded and the cells were rinsed with phosphate-buffered saline (PBS), then fixed with 4% paraformaldehyde (PFA) for 10 min at room temperature. The coculture system was evaluated using control NDCs cocultured with control OLs and FAHN NDCs cocultured with FAHN OLs.

### 2.7. Western Blot Analysis

Cells were dissociated using Accutase, and lysates were prepared by incubating the cells for 20 min on ice in RIPA lysis buffer. The lysis buffer contained 137 mM Tris-HCl, 12 mM NaCl, 2 mM sodium deoxycholate, and 0.1 mM EDTA and was supplemented with 0.1% SDS, 1% Triton^®^ X-100, 10% glycerol, a cOmplete™, Mini, EDTA-free Protease Inhibitor Cocktail, and PhosSTOP™ phosphatase inhibitor (both Roche Diagnostics GmbH, Mannheim, Germany). After lysis, the samples were centrifuged at 15,000× *g* for 20 min at 4 °C. Protein concentrations in the supernatants were determined using the Pierce™ BCA Protein Assay Kit (Thermo Fisher Scientific, Dreieich, Germany), following the manufacturer’s protocol. Samples were then heated for five minutes at 95 °C in Laemmli buffer (125 mM Tris, 20% glycerol, 2% SDS, 5% β-mercaptoethanol, and 10% bromophenol blue) before being centrifuged again at high speed. For electrophoresis, homemade 15% SDS-PAGE gels were prepared using 1.5 M Tris (pH 8.8), 10% SDS, 10% ammonium persulphate (APS), and 40% Roti^®^Phorese Gel 40 solution; 4% stacking gels were made using 0.5 M Tris (pH 6.8), 10% SDS, 10% APS, and 40% Roti^®^Phorese Gel 40 solution, all mixed with ultrapure water. The running buffer contained 250 mM Tris, 2 M glycine, and 0.1% SDS. Protein transfer was performed using the Trans-Blot^®^ Turbo™ Transfer System and Trans-Blot^®^ Turbo™ Transfer Pack (midi format, 0.2 μm nitrocellulose; Bio-Rad Laboratories, Hercules, CA, USA). Membranes were washed three times for five minutes each in Tris-buffered saline (TBS; 20 mM Tris, 137 mM NaCl, pH 7.5), then blocked for one hour in 5% bovine serum albumin (BSA) diluted in TBS containing 0.1% Tween^®^ 20 (TBST). Membranes were incubated overnight at 4 °C with primary antibodies (Table 1). Detection of GAPDH involved incubation with primary antibody diluted in 3% BSA/TBST for one hour at room temperature. After primary antibody incubation, membranes were washed three times for five minutes in TBST and then incubated for one hour at room temperature with DyLight™-conjugated secondary antibodies.

### 2.8. Immunofluorescence Staining

For immunofluorescence staining, cells were plated on PLO/laminin-coated glass coverslips at a density of 50,000 cells/cm^2^. Cells were rinsed with DPBS and fixed with 4% paraformaldehyde (PFA) for 15 min at room temperature. Following fixation, cells were washed three times with DPBS and then permeabilised using 0.2% Triton X-100 in DPBS for 15 min at room temperature. To block non-specific binding, cells were incubated with Pierce™ Protein-Free Blocking Buffer (Thermo Fisher Scientific, Dreieich, Germany) for one hour at room temperature. Primary antibodies (Table 1) were applied in blocking buffer and incubated overnight at 4 °C. The next day, cells were washed three times in DPBS for five minutes each. Secondary antibodies—including anti-rabbit IgG, anti-mouse IgG, anti-mouse IgM, and anti-chicken IgY conjugated to Alexa Fluor 488, 568, or 642—were diluted 1:1000 in blocking buffer and applied for one hour at room temperature in the dark. After three additional DPBS washes, cells were mounted using DAPI Fluoromount-G^®^ (SouthernBiotech, Birmingham, AL, USA) and stored at 4 °C. Imaging was performed on an LSM 900 laser scanning microscope (Zeiss, Hamburg, Germany) equipped with a motorised scanning stage, multi-wavelength laser module, high-resolution oil immersion objective, and GaAsP-PMT detector. Image acquisition and analysis were conducted using ZEN 2 Blue Edition software (Version 3.9). Colocalisation analysis was performed in ImageJ using the Just Another Colocalisation Plugin (JaCoP, ImageJ Plugin Repository, NIH Bethesda, MD, USA) to calculate Pearson’s correlation coefficient.

### 2.9. Statistical Analyses

Statistical analysis was performed using GraphPad Prism, version 8.0.1. Data are presented as mean ± standard deviation (SD), with each value derived from a minimum of six independent experiments to ensure robustness and reproducibility. Normality of datasets was assessed using the Shapiro–Wilk test. For comparisons between two groups, the unpaired Student’s *t*-test was used. When comparing more than two groups, a one-way analysis of variance (ANOVA) was performed. If significant differences were detected, Dunnett’s post hoc test was applied to identify pairwise differences relative to the control group. For non-normally distributed data, the Kruskal–Wallis test was used as a non-parametric alternative. In these cases, Dunnett’s post hoc test was also applied to assess significance between groups. A *p*-value of ≤0.05 was considered statistically significant. Significance levels are indicated as follows: *p* ≤ 0.05 (#, $), *p* ≤ 0.01 (##, $$), and *p* ≤ 0.001 (###, $$$).

## 3. Results

### 3.1. Establishing the Disease Model

**Figure 1:**
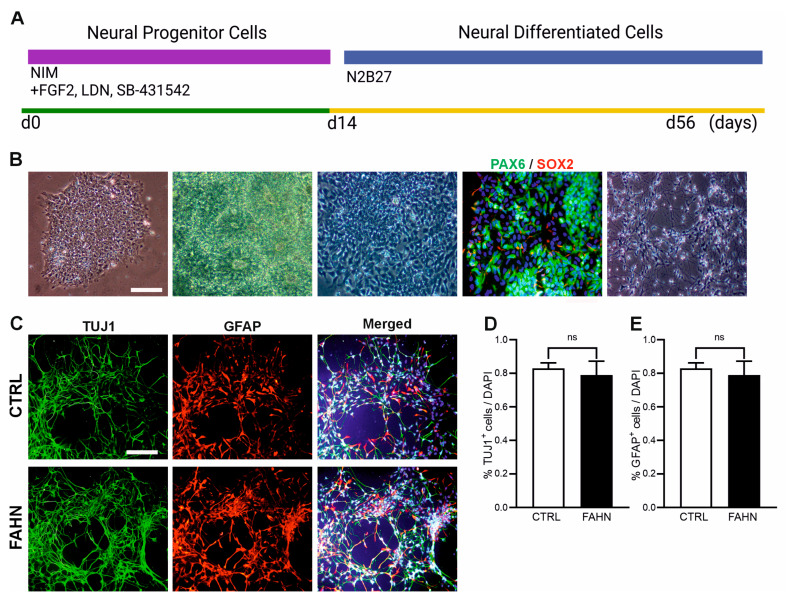
Characterisation of neural progenitor cells and neurally differentiated cells. (**A**) Schematic overview of the generation of neural progenitor cells (NPCs) and the subsequent differentiation into neuronal derivatives (NDCs). (**B**) hiPSCs were cultured as colonies on Matrigel-coated plates using a small-molecule-enriched medium, facilitating the formation of neural rosettes composed of PSA-NCAM^+^ NPCs. These progenitors were isolated by magnetic-bead-based purification. Immunofluorescence staining confirmed the expression of neural stem cell markers PAX6 (green) and SOX2 (red), with nuclei counterstained using DAPI (blue). N = 18, n = 3 independent differentiations per line. Scale bars: 100 μm (bright field), 20 μm (NPC immunofluorescence). NPCs were expanded and differentiated into NDCs over a six-week period. (**C**) NDCs derived from both the control (CTRL) and FA2H-deficient (FAHN) hiPSC lines displayed immunoreactivity for the neuronal marker TUJ1 (green) and the astrocyte marker GFAP (red). Nuclei were stained with DAPI (blue). (**D**,**E**) Quantification of TUJ1^+^ and GFAP^+^ cells revealed no significant differences between CTRL and FAHN lines, confirming that both generate a mixed culture of neurons and astrocytes. N = 20, n = 9 independent differentiations per line. Scale bar: 100 μm. ns = not significantly different.

The primary objective of this study was to identify and analyse the pathologies associated with disease in neurons and OLs that are specifically affected in FAHN mouse models. To achieve this, we made use of recently generated FAHN-patient-derived hiPSCs [23] and generated NPCs and neural derivatives, using small-molecule-based culture techniques (Figure 1A). Following the completion of culturing and neural rosette formation (Figure 1B), NPCs expressing the surface marker PSA-NCAM were isolated using magnetic beads (Figure 1B). These cells were further characterised for the presence of neuroectodermal stem cell markers including PAX2 and SOX2 (Figure 1B). A substantial majority of the cells demonstrated the expression of these markers, confirming their successful differentiation into NPCs. Subsequently, these NPCs were utilised as a platform for generating NDCs, which then underwent spontaneous differentiation over a period of six weeks (Figure 1C). The differentiated cells were found to robustly express the neuronal cell marker TUJ1 (green) and the astrocyte marker GFAP (red) (Figure 1C). The quantification confirmed the visual impression indicating that there is no significant difference between the FA2H-deficient cell line and the corresponding control cell line in differentiation of neuronal cells (Figure 1D,E). These results suggest that NPCs derived from hiPSCs are capable of differentiating into both neuronal and astrocytic cells, thus serving as a valuable in vitro model for studying neural differentiation and further disease-specific investigations.

**Figure 2:**
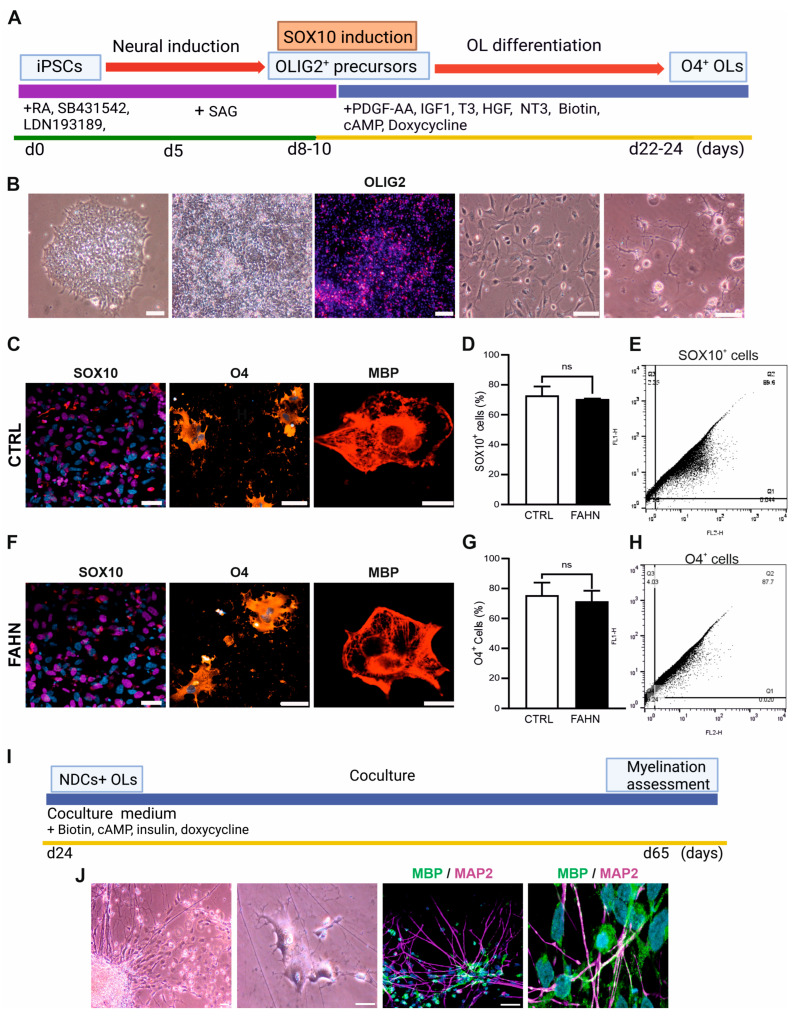
Generation and characterisation of oligodendrocytes and formation of myelinating cocultures. (**A**) Schematic representation of the key stages in the generation of oligodendrocytes (OLs) using a lentiviral transduction-based protocol. (**B**) hiPSC colonies were cultured on Matrigel-coated plates and treated with SMAD inhibitors for eight days to induce OLIG2^+^ precursors. These cells were subsequently transfected with the SOX10 transcription factor to promote oligodendrocyte differentiation. After ten additional days in culture mature OLs, displaying characteristic wide, branched morphology, became visible. Immunofluorescence analysis confirmed stage-specific expression of key markers in both the control (CTRL, (**C**)) and FA2H-deficient (FAHN, (**F**)) lines: OLIG2 at day 8 (pre-transfection), SOX10 at 24 h post-transfection, O4 (orange) during pre-myelinating stages, and MBP (red) at later stages, indicating myelin expression. Nuclei were counterstained with DAPI (blue). n = 20 independent differentiations per line. Scale bars: 100 μm, 50 μm (OLIG2 and SOX10), 20 μm (MBP). (**D**) Quantitative analysis of SOX10^+^ and O4^+^ cells showed no significant differences between CTRL (**D**) and FAHN lines (**G**). Representative FACS dot plots (**E**,**H**) showing the percentage of O4^+^ cells 10 days after SOX10 induction. Debris was excluded by forward and side scatter, followed by gating for single, live cells. OL identity was defined by O4 positivity. Flow cytometry analysis was performed using FlowJo software (version 10). n = 2. Brightfield and OLIG2: scale bar = 150 μm; SOX10 = 100 μm; O4 = 50 μm; MBP = 20 μm. n = 10 per differentiation, n = 20 independent differentiations per line. (**I**) Mature OLs and neurons were combined in coculture and maintained for six weeks. At 24 h, OLs adhered to neurons, and by day 4, they began extending processes and wrapping axons. (**J**) Immunostaining after four days of coculture revealed MBP^+^ myelin (green) surrounding MAP2^+^ neurons (magenta). Nuclei were counterstained with DAPI (blue). Scale bars: 50 μm and 20 μm. n = 13, n = 9 independent differentiations per line. ns = not significantly different.

Generation of OPCs was performed using hiPSCs as a starting point (Figure 2A). The process of neural induction was meticulously carried out over a period of eight to ten days through the application of several small molecules (SB431542 and LDN193189, RA, and SAG) that inhibit SMAD signalling pathways, resulting in the formation of neural rosettes (Figure 2B). This precise combination of molecules facilitated a significant increase in the percentage of OLIG2^+^ cells. During the second stage of OPC generation, cells were transduced with a lentiviral vector encoding the SOX10 coding sequence. Following a 24 h period post-transfection, a majority of the cells displayed a notable change in morphology, exhibiting characteristics typical of pre-OPCs. Cells were found to be positive for SOX10 (Figure 2C,F, red). In the ultimate stage of OPC generation, cells were cultured in an OL differentiation medium supplemented with 1 μg/mL doxycycline to promote SOX10 expression, until day 24. The morphological characteristics of these OLs included an enlarged, roundish nucleus along with extensive external branches and dendrites, positively expressing O4 (orange) and MBP (red) (Figure 2C,F). The OPCs can be purified by their expression of both PSA-NCAM and O4, using MicroBeads. The quantification of SOX10-positive cells shows there is no difference in expression of SOX10 (Figure 2D,E) and O4 (Figure 2G,H) in the FA2H-deficient cell line and control cell line, respectively. At the conclusion of this differentiation stage, approximately 55–70% of the cells were found to express O4, while around 30% demonstrated expression of the myelin marker MBP, indicating mature OLs. Considering that a key hallmark of FAHN disorder involves demyelination and myelin instability [2], we utilised in our subsequent experiments oligodendrocytes and cocultures derived from FAHN and control iPSCs. The coculture experiments were performed in accordance with the following conditions: control NDCs + control OLs and FAHN NDCs + FAHN OLs (Figure 2I). Myelination depends on proper interaction with the substrate. Thus, we co-cultured OLs with human iPSC-derived neurons to further investigate putative myelination impairments in the case of FAHN. Initially, during the culturing process, maturated OLs began to form intricate networks and to engage in wrapping around axons (Figure 2J). To achieve a fully myelinating model, purified OLs from day 24 and neurons from day 56 were cultured for a period of six weeks. This protocol resulted in a highly integrated oligodendrocyte–neuronal myelinating coculture, which was subsequently verified through immunostaining employing antibodies against MBP (green) to stain myelin and antibodies against MAP2 (magenta) to stain neurons (Figure 2J).

### 3.2. FA2H Mutant Protein Is Expressed at Reduced Levels in Patient-Derived Cells

**Figure 3:**
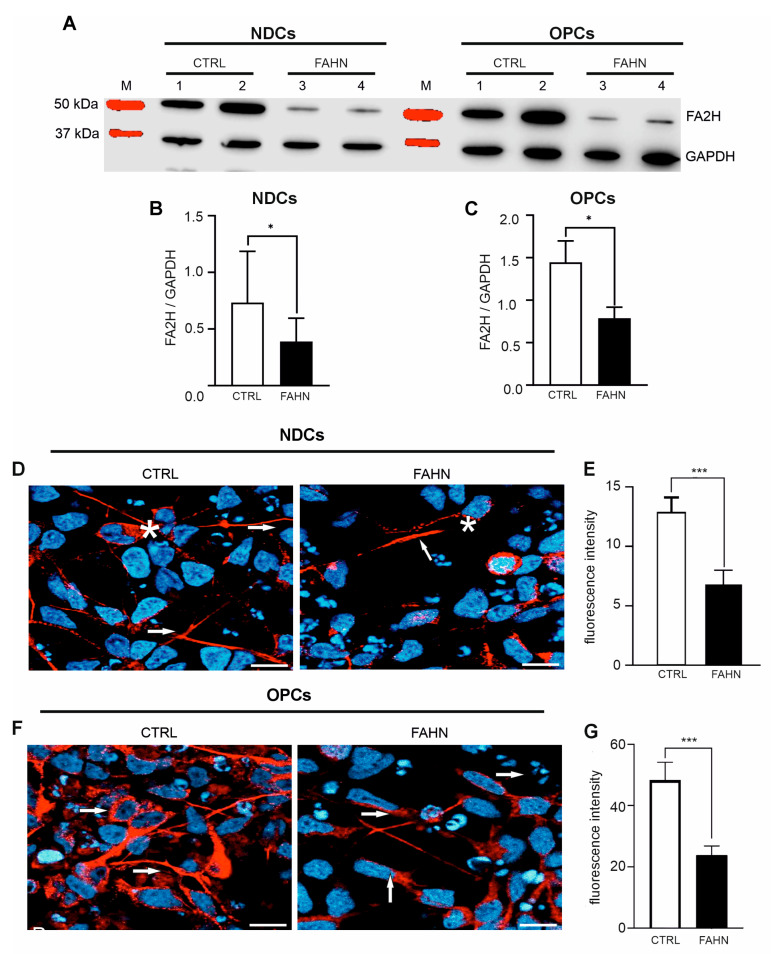
FA2H protein expression and intracellular localisation of iPSC-derived neuronal and oligodendrocyte cells. (**A**) Western blot analysis was conducted to determine total FA2H protein levels in neurally differentiated cells (NDCs) and oligodendrocyte precursor cells (OPCs). Band intensities for FA2H were normalised to GAPDH, which served as the reference protein. (**B**,**C**) Quantification of Western blot signals revealed a significant reduction in total FA2H protein levels in FA2H-deficient NDCs and OPCs compared to controls (N = 12), indicating impaired FA2H expression in the mutant cell lines. (**D**,**E**) Intracellular localisation of FA2H protein was examined in NDCs (**D**) and OPCs (**E**) using immunofluorescence staining (FA2H in red; nuclei counterstained with DAPI (blue). In control NDCs, FA2H was strongly expressed in the somata (marked by *) and neuritic projections (indicated by arrows). In contrast, FA2H-deficient NDCs displayed markedly reduced fluorescence and a diffuse intracellular distribution. In OPCs, FA2H in control cells appeared as intense cytoplasmic puncta near the nucleus (arrows), while deficient cells exhibited weaker yet similarly distributed signals. (**F**,**G**) Quantitative fluorescence analysis confirmed a significant reduction in FA2H signal intensity across all FA2H-deficient cell types (N = 16), supporting the Western blot results. Scale bars: 20 μm. *p* < 0.05 (*), *p* < 0.001 (***) by one-way ANOVA followed by Dunnett’s post hoc test compared to CTRL. NDCs = neurally differentiated cells; OPCs = oligodendrocyte-differentiated cells; M = size marker.

To analyse the impact of mutations on FA2H protein expression, Western blot analyses were conducted on FA2H-deficient and control cells. Representative images (Figure 3A,C) depict the FA2H band pattern in both NDCs and OPCs cells from FA2H-deficient cells. The total FA2H protein was identified as a distinct band at approximately 50 kDa in both NDCs and OPCs, visually suggesting a weak signal in the FA2H protein. Furthermore, quantification revealed a significantly decreased signal of total FA2H protein in the FA2H-deficient NDCs or OPCs compared to the control NDCs or OPCs (Figure 3B,C), indicating that mutations in the *FA2H* gene contribute to reduced steady-state levels of the endogenously expressed FA2H protein. Intriguingly, the total expression of FA2H protein in OPCs was higher than in NDCs, suggesting a higher requirement and expression of FA2H protein in oligodendrocytes. These findings collectively indicate that the expression of FA2H protein in both types of FA2H-deficient cells is significantly reduced, yet it is markedly elevated in oligodendrocyte-differentiated cells, underlining the differential demand for FA2H protein between neuronal and oligodendrocyte differentiation.

### 3.3. FA2H Mutant Protein Displays Incorrect Intracellular Localization in Patient-Derived Cells

Investigations on FA2H proteins have revealed variances in protein levels between FA2H-deficient cells and control cell lines. This prompted a study of whether mutant FA2H protein retains its typical cellular location in NDCs (Figure 3D) and OPCs (Figure 3F). We employed immunofluorescence staining of FA2H proteins and confocal microscopy to track its intracellular distribution. The intensity of FA2H fluorescence was quantified to gauge expression levels. In control neuronal cells, FA2H prominently localised to somata, dendrites, and axons, exhibiting strong fluorescence. However, mutant cells displayed weaker, diffuse staining, indicating disrupted localisation. Specifically, in NDCs of the FA2H-deficient cell line, there was a notable relocation of FA2H from somata to axonal areas, with minimal intracellular presence. Quantitative analysis supported these observations, with control cells showing high fluorescence intensities indicative of abundant cytoplasmic FA2H (Figure 3E,G). Conversely, the FA2H-deficient cells exhibited markedly lower fluorescence, suggesting reduced cytoplasmic protein presence. In oligodendrocyte-differentiated cells, control samples showed intense FA2H signals as cytoplasmic clusters near the nucleus. In contrast, FA2H-deficient OPCs displayed weaker signals but maintained a similar distribution. Comparative fluorescence intensity analysis further confirmed lower FA2H levels in deficient OPCs compared to control OPCs. Overall, our data reveal multiple cellular abnormalities in FAHN, highlighting diminished FA2H protein levels and altered expression in mutant proteins in both neuronal and oligodendrocyte cells.

### 3.4. MBP and d PLP Are Expressed at Reduced Levels in FA2H-Deficient Cells

**Figure 4:**
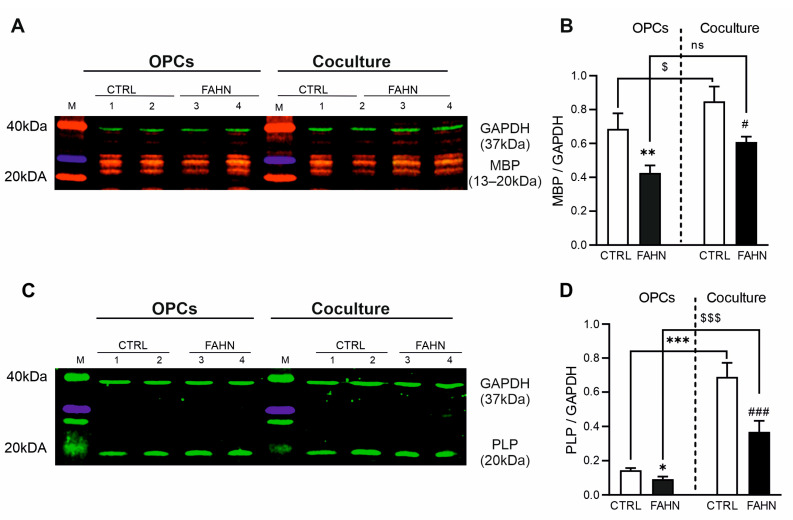
Expression of myelin proteins in FA2H-deficient cell line. Western blot analysis of myelin basic protein (MBP, (**A**)) and proteolipid protein (PLP, (**C**)) in oligodendrocyte progenitor cells (OPCs) and cocultures. Samples were taken from four replicates each of OPCs and cocultures (lanes 1–4). GAPDH (37 kDa) was used as a loading control. MBP was detected at approximately 1320 kDa. FA2H-deficient OPCs and cocultures exhibited lower MBP expression, whereas increased MBP expression was observed only in cocultures of the control cell line. (**B**) Quantification confirmed a significant reduction in MBP levels in both FA2H-deficient OPCs and cocultures compared to controls. (**C**) PLP expression was assessed by Western blot, with the main band detected at 20 kDa in both OPC and coculture samples. Control cell lines showed stronger PLP signals in both conditions compared to FA2H-deficient cells. (**D**) Quantification revealed significantly reduced PLP levels in FA2H-deficient OPCs and cocultures relative to controls. Interestingly, PLP levels were significantly higher in cocultures compared to their respective OPC monocultures in both cell lines. N = 14–16. *p* < 0.05 (*, #, $), *p* < 0.01 (**), *p* < 0.001 (***, ###, $$$) by one-way ANOVA followed by Dunnett’s post hoc test compared to CTRL. OPCs = oligodendrocyte progenitor cells; M = size marker.

These studies address the severe neurodegenerative symptoms and notable demyelination observed in FAHN patients. We began by evaluating the expression of myelin’s most abundant proteins, MBP and PLP, within OPCs and cocultures using Western blot methodology (Figure 4). For effective myelination and axonal wrapping, it is crucial that OLs produce adequate and comparable amounts of MBP, PLP, and other structuring proteins. A comprehensive evaluation of the MBP (Figure 4A) revealed the presence of a distinct and well-defined band with an estimated molecular weight of approximately 13–20 kDa in the control OPC line. This result indicates an elevated level of expression. In contrast, the FA2H-deficient cell line displayed a visually reduced MBP signal. This decrease was confirmed quantitatively, showing significantly lower MBP levels in oligodendrocyte-differentiated cells of the mutant line (Figure 4B). Additionally, a similar analysis was conducted on the cell-based in vitro coculture consisting of neurons and myelinating oligodendrocytes, which provided insights into compact myelin formation (Figure 4A,B). The expression of MBP in the coculture of the control cell line showed a strong and bright signal, indicative of an increased amount of MBP. However, in the patient coculture cells, a reduced level of MBP was observed, similar to that seen in the oligodendrocyte cells, and was visually lower compared to the control line (Figure 4B). Collectively, these results indicate a reduced expression of MBP in both oligodendrocyte and coculture cells of the mutant line, corroborating previous reports in mice models that have shown marked reductions in MBP in the central and peripheral nervous system [19,20]. The FA2H-deficient cell line consistently demonstrated a reduced level of MBP in both oligodendrocyte and coculture cells compared to the control. Next to our MBP findings, we evaluated the expression of PLP using Western blot analysis (Figure 4C,D). The results showed significantly reduced PLP levels in the FA2H-deficient OPCs compared to the corresponding control line. Similarly, coculture of FA2H-deficient cells also displayed significantly reduced PLP levels, confirming the impaired production of myelin membrane in FA2H-deficient cells. Overall, our data demonstrate significant impairments in myelin protein synthesis in FA2H-deficient hiPSC-derived OPCs and cocultures and reveal enhanced protein expression in cocultures, where OPCs interact with neurons.

### 3.5. FA2H Mutant OPCs and Coculture Cells Show Impaired Localisation of MBP and PLP

**Figure 5:**
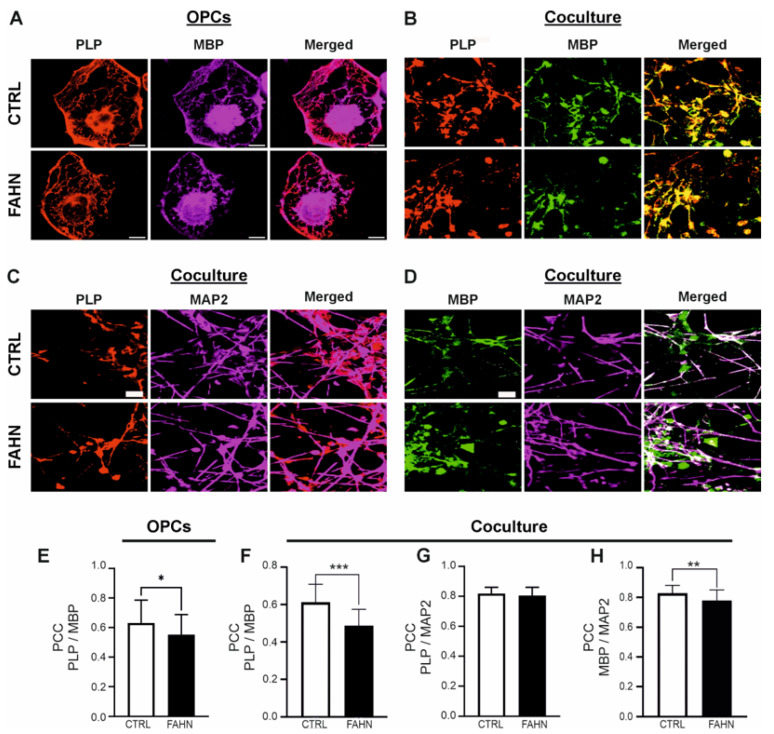
Colocalisation analysis of MBP and PLP in OPCs and cocultures. (**A**) Representative confocal image showing immunofluorescence staining for PLP (red) and MBP (magenta) in OPCs. In the control cell line, PLP was strongly expressed and predominantly localised to myelin extensions and perinuclear regions, with substantial colocalisation with MBP. In contrast, OPCs derived from the FA2H-deficient cell line exhibited a markedly reduced PLP signal and minimal colocalisation with MBP. (**B**) Confocal images of coculture samples stained for PLP (red) and MBP (green). In the FA2H-deficient line, PLP expression was visibly reduced, accompanied by a pronounced reduction in colocalisation with MBP in the myelin sheaths formed by oligodendrocytes and neurons. (**C**,**D**) Confocal microscopy images showing localisation of PLP (red), MBP (green), and MAP2 (magenta) in cocultures. The PLP/MAP2 fluorescence signals were strongly colocalised in both control and FA2H-deficient lines, particularly along axonal segments where myelin forms. (**E**,**F**) Quantification of Pearson’s correlation coefficient (PCC) confirmed reduced PLP/MBP colocalisation in all FA2H-deficient cell types, indicating impaired integration of these proteins into myelin structures. (**G**) Quantitative evaluation of PLP/MAP2 colocalisation revealed no significant difference between the FA2H-deficient and control lines. Both demonstrated similarly high PCC values. (**H**) MBP/MAP2 colocalisation was significantly reduced in the FA2H-deficient cell line. While control cells showed robust colocalised fluorescence along axons, the FAHN cell line exhibited a weaker signal, with MBP localisation concentrated primarily in somata. Scale bars: 20 μm (A–D), 10 μm (E–H). N = 49 OPCs, N = 38–40 cocultures. *p* < 0.05 (*), *p* < 0.01 (**), *p* < 0.001 (***) by paired *t*-test compared to CTRL. OPCs = oligodendrocyte progenitor cells.

To better understand the localisation and interaction of PLP (red) and MBP (magenta) in OPCs and myelinating cells in coculture, we performed colocalisation analyses and quantified the extent of overlap using Pearson’s correlation coefficient (PCC) (Figure 5). We first examined PLP/MBP colocalisation in individual OPCs. In the control cell line, fluorescence signals for PLP and MBP showed considerable overlap, resulting in pinkish staining in cellular processes, myelin extensions, and perinuclear regions—areas typically characterised by extensive plasma membrane sheets (Figure 5A, upper panel). In contrast, OPCs derived from the FA2H-deficient cell line displayed weak colocalisation, with PLP and MBP signals largely confined to cell processes and myelin extensions (Figure 5A, lower panel). Quantitative analysis confirmed these observations: control cells exhibited high PCC values, indicative of strong colocalisation and coordinated localisation of PLP with MBP. FA2H-deficient OLs, however, showed significantly lower PCC values, reflecting impaired protein colocalisation within the myelin compartments (Figure 5E). A similar pattern was observed in coculture experiments. Control cocultures showed robust overlap of PLP and MBP fluorescence signals, whereas the FA2H-deficient cocultures exhibited weaker and more diffusely distributed staining (Figure 5B). Quantification again revealed significantly reduced PCC values in FA2H-deficient cocultures compared to controls (Figure 5F), supporting the notion of disrupted myelin protein organisation in FAHN.

### 3.6. Mutant FA2H Cells Line Has Reduced Quantity of Myelin Proteins

Based on previous studies and the findings of this investigation, it is evident that cells harbouring mutated FA2H are capable of synthesising myelin sheaths and forming myelin membranes; however, these membranes are structurally unstable and lack proper compaction [2,13,19,20]. To evaluate the extent of axonal myelination, we hypothesised that the reduced expression and colocalisation of PLP and MBP would lead to a decrease in the number of myelinated axons in FA2H-deficient cocultures. We began by analysing the colocalisation of PLP and MBP with the neuronal marker MAP2 (Figure 5C,D). In both control and FA2H-deficient cocultures, PLP showed robust expression and strong colocalisation with MAP2 in neuritic regions, suggesting that aspects of myelin membrane formation were preserved in the mutant cells (Figure 5C). By contrast, MBP fluorescence in control cells was primarily localised along neurites and showed extensive overlap with MAP2 staining (Figure 5D). In FA2H-deficient cells, however, MBP was less colocalised with MAP2, suggesting impaired integration of MBP into axonal segments. Quantification of colocalisation in OPCs revealed significantly lower PCC values for PLP/MBP in FA2H-deficient cells compared to controls (Figure 5E). A similar trend was observed in cocultures, where PLP/MBP colocalisation remained reduced in the FA2H-deficient line (Figure 5F), indicating compromised localisation of myelin proteins under myelinating conditions. Interestingly, colocalisation of PLP with MAP2 remained comparably high in both FA2H-deficient and control cocultures (Figure 5G), whereas MBP/MAP2 colocalisation was markedly lower in the FA2H-deficient cells. This was further supported by PCC analysis, which revealed a significant reduction in MBP/MAP2 overlap in the mutant line (Figure 5H).

Collectively, these results demonstrate that PLP and MBP show impaired colocalisation in FA2H-deficient iPSC-derived oligodendrocytes and cocultures, reflecting disrupted myelin protein integration. These defects point to an imbalance in myelin homeostasis and reveal compromised membrane architecture in FAHN pathology. The data underscore the critical link between protein mislocalisation and structural abnormalities in myelin formation caused by FA2H mutation.

### 3.7. Myelin Sheath Examination and Ranvier Nodes

**Figure 6:**
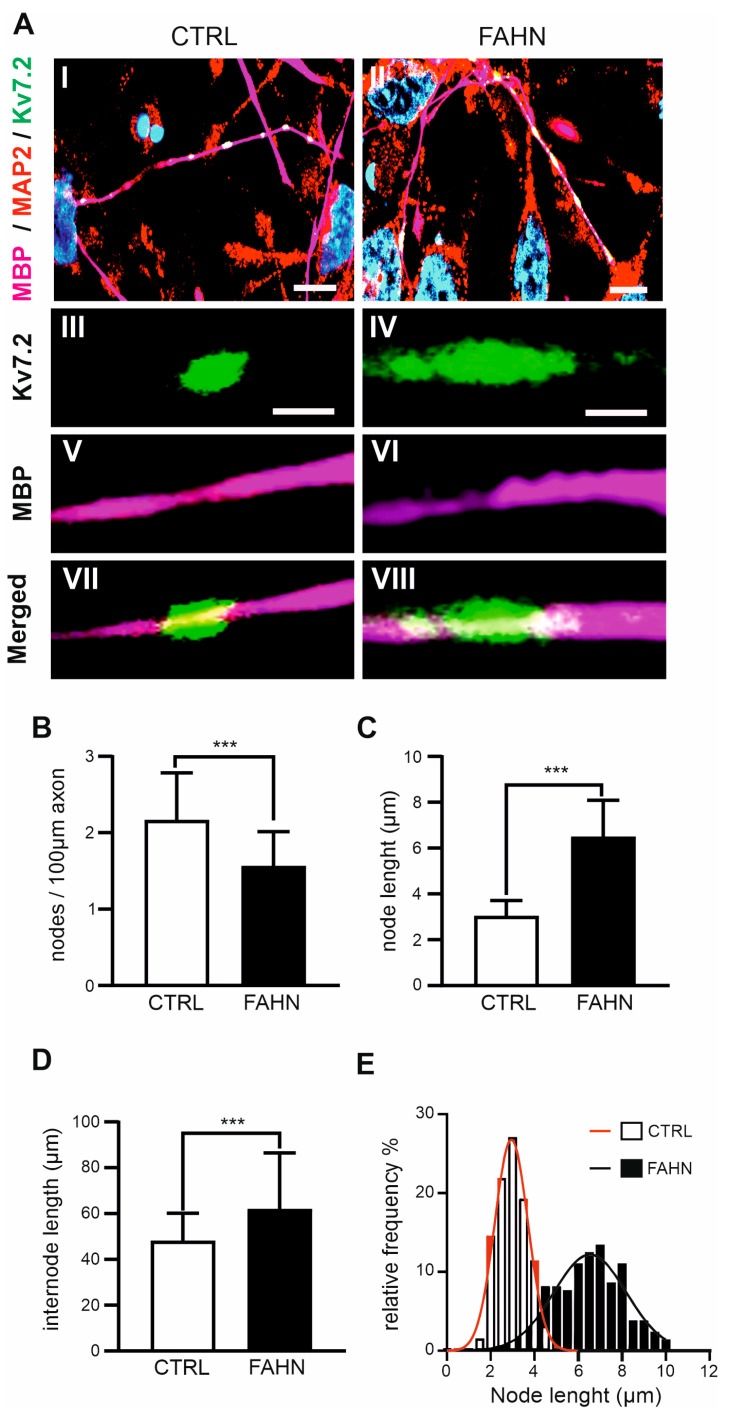
Analysis of Kv7.2 channel expression in nodes of Ranvier. (**A**) Representative confocal images of control (CTRL, **AI**) and FA2H-deficient cell line (FAHN, AII) showing nodes of Ranvier, labelled with Kv7.2-positive clusters (green), myelinated axons marked by MBP (magenta), and axons stained for MAP2 (red). Scale bar: 20 µm. Magnifications of Kv7.2 cluster (AIII,AIV), MBP signal (AV,AVI), and merged signals (AVII,AVIII) of control cells (CTRL) and FA2H-deficient cells (FAHN). Both cell lines display strong Kv7.2 fluorescence at nodal regions and along internodes, indicating the presence of myelinated axons. (**B**) Quantification of the number of nodes per 100 µm of axon confirms that the FA2H-deficient cell line exhibits fewer, but longer, nodes of Ranvier (**C**). N = 40. (**D**) Quantification of internode length shows a significantly increased mean internodal length in the FA2H-deficient cell line compared to the control. N = 40. (**E**) Frequency distribution of node lengths with fitted Gaussian curves for the control (red) and FAHN (black) groups, demonstrating greater heterogeneity in node length in FA2H-deficient axons. N = 40. Gaussian fits of node length distributions: CTRL: A = 26.74 µm, mean (M) = 2.945 µm, r = 0.9878 µm. FAHN:A = 12.19 µm, M = 6.540 µm, r = 1.337 µm. N = 192–207; values are mean ± SD. *p* < 0.001 (***) versus CTRL by two-sided Kolmogorov–Smirnov test.

In the next stage of analysis, we investigated myelinated axons using the previously described coculture system of iPSC-derived oligodendrocytes (OLs) and neurons from the FA2H-deficient cell line. The structural integrity of myelinated axons is essential for fast, energy-efficient signal propagation, which depends on precise subcellular organisation of voltage-gated ion channels. This study therefore focused on assessing the length, number, and localisation of nodes of Ranvier and internodes. To visualise these structures, we employed immunofluorescence labelling of nodal and juxtaparanodal proteins. This allowed us to identify myelinated axons, trace individual fibres, and determine the spatial arrangement of nodes and internodes. Specifically, the potassium channel Kv7.2 was used to label nodes of Ranvier, MBP to identify myelinated axons, and MAP2 to stain neurites (Figure 6A). In the control cell line, myelinated axons displayed clearly defined nodal structures characterised by clustered accumulations of Kv7.2. By contrast, the FA2H-deficient cells exhibited abnormal morphologies, with irregular node-like formations and a mix of short and elongated internodes (Figure 6A). Quantitative analysis supported these observations. We quantified the number of nodes per 100 µm of axon across 40 axons. FA2H-deficient cells had significantly fewer nodes (1.55 per 100 µm) compared to controls (2.16 per 100 µm) (Figure 6B). The average internode length in FA2H-deficient cells (63.8 ± 4.28 µm, MBP-positive fibres) was significantly longer than in the control cell line (47.9 ± 2.61 µm) (Figure 6C). Visual inspection also revealed striking differences in node morphology between the two lines. In FA2H-deficient axons, node lengths were broadly distributed, ranging from 2.22 to 9.98 µm, significantly wider than in control cells (2.53 to 4.86 µm) (Figure 6D,E).

In summary, the FA2H-deficient cell line displayed disrupted myelinated axonal architecture, characterised by irregular internode lengths and reduced, disorganised node density. Some axons exhibited dense clustering of nodes, while others had only one or two nodes per 100 µm. Moreover, the nodes themselves appeared larger and less compact, with broader Kv7.2 staining patterns—suggesting impaired node formation and maintenance in the absence of functional FA2H.

### 3.8. Initiation and Progression of Autophagy Is Affected

**Figure 7:**
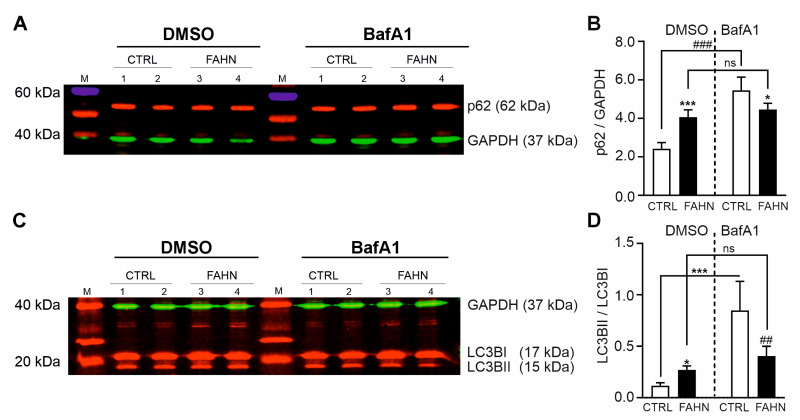
Expression of autophagy markers p62 and LCB in NDCs. (**A**) Representative Western blot images showing expression of p62 under basal conditions (DMSO) and following treatment with bafilomycin (BafA1). GAPDH (37 kDa) served as a loading control. p62 protein (62 kDa) was detected in neurally differentiated cells (NDCs) under basal conditions (lanes 1–4) and after BafA1 treatment (lanes 1–4). In the FA2H-deficient cell line, the p62 band showed a markedly stronger signal compared to the corresponding control, indicating elevated expression. (**B**) Quantification under basal conditions confirmed a significant increase in p62 protein in the FA2H-deficient cell line compared to untreated controls. Following six hours of BafA1 treatment, only the control cell line showed a further increase in p62 levels, whereas the FA2H-deficient line did not respond similarly. N = 16. (**C**) Representative Western blot showing LC3B expression under basal and BafA1-treated conditions. LC3BI and LC3BII were detected at approximately 17 kDa and 15 kDa, respectively. In the FA2H-deficient cells, strong LC3BI and LC3BII bands were observed under both conditions. (**D**) Quantification revealed a significantly increased LC3BII/LC3BI ratio under basal conditions in the FA2H-deficient cell line compared to untreated controls. After BafA1 treatment, only the control cells showed increased LC3BII levels, while the FA2H-deficient cells exhibited no further increase—mirroring the response pattern seen for p62. N = 12. *p* < 0.05 (*), *p* < 0.01 (##), *p* < 0.001 (***, ###), ns = not significantly different, by one-way ANOVA followed by Dunnett’s multiple comparisons test.

As previously reported, autophagy is impaired in FA2H-deficient cells [21]. To expand on this observation, we conducted a comprehensive analysis focusing on the initiation and progression of autophagy, particularly the formation of autophagosomes and the sequestration of cargo destined for degradation. To evaluate autophagosome formation in FA2H-deficient neurally differentiated cells (NDCs), we performed Western blot experiments targeting two key autophagy markers: sequestosome-1 (p62/SQSTM1) and LC3B (Figure 7A–D). Our initial analysis focused on p62, a ubiquitin-binding scaffold protein that links polyubiquitinated cargo to LC3BII, facilitating their engulfment into autophagosomes. Under basal conditions, the control cell line exhibited a stable p62 signal, whereas the FA2H-deficient cell line showed a markedly stronger band, indicating elevated p62 protein levels (Figure 7A). Quantification confirmed this increase, revealing significantly higher p62 expression in FA2H-deficient cells compared to controls (Figure 7B). To probe autophagy initiation, we inhibited autophagosome–lysosome fusion by treating cells with bafilomycin A1 (BafA1) for 6 h. BafA1 blocks vacuolar H^+^-ATPase activity, preventing acidification and lysosomal fusion, which leads to accumulation of autophagosomes and their cargo. Following BafA1 treatment, control cells showed a substantial increase in p62 levels, as expected (Figure 7B). Interestingly, in the FA2H-deficient cell line, p62 levels remained unchanged after BafA1 treatment, suggesting a defect in cargo degradation or trafficking into the autophagic pathway. Next, we analysed LC3B, a widely used marker of autophagosomes. We compared the LC3BII/LC3BI ratio under basal and BafA1-treated conditions (Figure 7C,D). In control cells, both LC3B isoforms were expressed at moderate levels, with an increase in LC3BII observed upon BafA1 treatment. In contrast, FA2H-deficient cells displayed strong LC3BI and LC3BII signals under basal conditions, which were not further enhanced by BafA1 (Figure 7C). Quantification confirmed an elevated LC3BII/LC3BI ratio in the mutant line under both conditions (Figure 7D). These results suggest that FA2H-deficient cells may exhibit a saturation in autophagosome synthesis, with no further accumulation possible upon inhibition of autophagosome degradation. This points to a dual impairment: increased autophagosome production under resting conditions and defective clearance of autophagic vesicles, indicating a fundamental disruption of autophagic flux in FAHN pathology.

## 4. Discussion

This study aimed to characterise the pathophysiological mechanisms of FAHN by employing neurons and oligodendrocytes derived from patient-specific human iPSCs recently generated in our laboratory. These cells, bearing clinically confirmed pathogenic *FA2H* mutations in a native gene dosage, provide a human model system to study myelination processes and the effects of FA2H deficiency in developmentally mature neurons and myelinating glial cells. A coculture system of oligodendrocytes and neurons was established to investigate demyelination and myelin instability—hallmarks of central and peripheral nervous system (CNS and PNS) pathology in FAHN. To date, there is no standardised protocol for the differentiation of stem cells into myelinating human FAHN cells. The coculture protocol used in this study proved highly efficient, yielding homogeneous populations of oligodendrocytes and neurons. The main aims of this study included the investigation of FA2H localisation and expression, structural analysis of myelin and key myelin proteins, and exploration of autophagic mechanisms.

FA2H is primarily expressed in myelinating cells of the nervous system [2,19]. Its expression increases post-natally, alongside the upregulation of oligodendrocyte-specific genes such as MBP, PLP, and CGT, which are involved in myelin sheath synthesis [2,25]. This correlates with a rise in hydroxylated fatty acid (HFA) levels during brain development. *FA2H*^−/−^ mice show reduced levels of both GalC and HFA-sulphatides in brain and peripheral nerves [13]. Consistent with these findings, our hiPSC-derived neural and oligodendrocyte precursor cells (NDCs and OPCs) exhibited markedly reduced FA2H expression, further supporting the hypothesis that FA2H deficiency results in decreased myelin-associated protein expression. The mutant cell line showed low signal intensity and weak localisation of FA2H, in agreement with prior reports. Age-related FA2H downregulation has also been observed in various brain regions of knockout mice [2,19,20,26]. Interestingly, FA2H levels increased in 18-month-old FA2H^−/−^ mice before declining again with age [19,20], reinforcing the idea that FA2H expression is both developmentally regulated and essential for myelin maintenance.

Alderson et al. [2] reported low FA2H protein levels in microglia, with no detectable expression in primary astrocyte cultures. However, FA2H expression has also been documented in non-neural tissues, including kidney and keratinocytes [1,13,27,28]. In animal models, FA2H mutations lead to demyelination, myelin instability, and axonal degeneration, particularly in older animals, despite normal oligodendrocyte differentiation and myelin formation during early development [19]. Mice examined at four weeks or five months of age exhibited normal ultrastructure of myelin in both the CNS and PNS, with normal nerve conduction velocities [19,20]. However, at 18 months, the mice displayed axon degeneration, followed by myelin degeneration in the spinal cord and sciatic nerves [19,20,28]. This suggests that FA2H activity is not essential for myelin formation but is vital for the long-term maintenance of myelin. It is important to emphasise that, regardless of age, all existing animal models (mice and Drosophila) exhibit physiological symptoms closely resembling those observed in human FAHN patients [7,8,9,10,11,19,20,21,28]. These models display reduced physical activity, impaired coordination, and feeding difficulties, with symptoms progressively worsening with age.

In order to gain a deeper insight into the myelin structure in the FA2H-deficient cell line, an analysis was conducted on the most abundant myelin-building proteins, MBP and PLP. As previously reported, FA2H, MBP, and PLP are activated almost simultaneously during the differentiation process in OPCs in order to form the myelin membrane compositions [23,29]. The present study demonstrates that the myelin proteins PLP and MBP, which exhibit specific subdomain localisation within the myelin sheath, display impaired localisation and reduced expression levels in FA2H-deficient OPCs. It was hypothesised that this impairment in the synthesis and localisation of MBP and PLP was due to the lack of other myelin-structuring proteins expressed when OPCs interact with neurons. The aim of this study was to investigate possible rearrangements of lipid domain organisation in the oligodendroglial membrane using oligodendrocytes cultured in the presence of neurons. Indeed, coculture with neurons resulted in a notable increase in raft clustering within the oligodendroglial membrane, accompanied by a subsequent rise in protein levels. In accordance with the findings of other studies [2,19,20,28], our investigation revealed a notable reduction in the colocalisation and expression levels of PLP and MBP in the mutant cell line. Furthermore, there was a marked decrease in the colocalisation of MBP with the neuritic marker MAP2. The late-onset axonal degeneration observed in *FA2H*^−/−^ aged mice has also been described in other mouse mutants deficient in myelin-specific genes, including PLP [30] and CNP [31]. The absence of PLP in oligodendrocytes in mice has been demonstrated to result in early impairment of anterograde and retrograde transport in the respective axon, which is associated with late-onset axonopathy [29,32]. Impaired bidirectional signalling between neurons and oligodendrocytes may affect neuronal activity, potentially leading to reduced activity in *FA2H*^−/−^ mice despite the presence of structurally and functionally normal myelin [19,20]. Recent studies with membrane monolayers of a composition similar to the myelin outer leaflet showed that the size and number of membrane domains formed by GalC were significantly affected by 2-hydroxylation [31]. This could potentially influence signalling between myelinating cells and neurons that depend on lipid rafts. It has been demonstrated that a negatively charged protein, MBP, which is located in the inner leaflet of the lipid bilayer, binds to GalC and PLP in the outer leaflet to form lipid rafts [29]. However, the reduced presence of MBP may not be sufficient to induce lipid aggregation in the absence of a neuronal environment, which appears to be required for the condensation of MBP broadly expressed in the oligodendroglial membrane. In young and aged *FA2H*^−^/^−^ mice, which specifically lack the 2-hydroxylated species of sphingolipids, a slight reduction in sulphatide levels in peripheral nervous system (PNS) myelin has been observed [28]. Similarly, in a sulfotransferase knockout mouse model, which lacks a key enzyme involved in the synthesis of sulfoglycolipids, subtle structural abnormalities of the paranodal and nodal regions were reported, highlighting the essential role of glycolipids in maintaining proper nodal architecture in the central nervous system (CNS) [33]. In contrast, another study of complete *FA2H*^−/−^ mice reported no alterations in paranodal structure, spacing, or length in animals up to six months of age [19]. Nonetheless, it remains possible that such structural changes may emerge at later stages of disease progression but were not captured in that study. Unlike the preserved paranodal structures described in these previous models [19,28], our findings demonstrate that both internodal and nodal structures are disrupted, indicating a more widespread impairment of nodal organisation. We propose that nodal elongation and altered conduction velocity represent additional pathological features of FA2H deficiency, occurring alongside demyelination and axonal degeneration.

The identification of compromised myelination in FAHN [2,6,14,19,20,24] prompted us to investigate autophagy processes. It has been demonstrated that mutation in FA2H is affecting some of the main proteins responsible for material degradation in autophagy [21,34]. In a Drosophila model lacking FA2H, alterations were observed in mitochondrial fission and fusion, which affected the mitochondrial network [21]. Additionally, there was an increase in the levels of the autophagy marker LC3B and its activation. The inhibition of FA2H has been demonstrated to impair the fusion of autophagosomes and lysosomes [21]. In the case of FAHN, elevated levels of LC3BII have been linked to an increased number of autophagosomes, which can lead to the impaired clearance of autophagic vesicles [21]. In addition to elevated levels of LC3BII (see Figure 7), we observed altered expression of p62 (see Figure 7), which suggests the accumulation of LC3BII. The fusion of autophagosomes and lysosomes is a vital step in the degradation process, as autophagosomes alone are unable to degrade the cargo. During the induction of autophagy, cytosolic LC3BI undergoes a conversion to LC3BII, which subsequently incorporates into the autophagosomal membrane [35,36]. The levels of LC3BII in the autophagosomal membrane may influence the fusion process with lysosomes. An increase in LC3BII levels may be indicative of a deficiency in the recycling process, whereby LC3BII is returned to cytosolic LC3BI. The altered activity of autophagy-related protein 4 (ATG4), which is responsible for converting LC3B isoforms, results in a reduction in the fusion of organelles due to elevated LC3BII levels [37]. Another protein associated with mitophagy/autophagy, p62, serves as a targeting protein for cargo destined for degradation via autophagy. In FA2H-deficient cells, we observed a reduction in the efficacy of p62 expression when autophagic vesicle (AV) clearance was impeded by BafA1 (Figure 7C,D), a finding that was consistent with the results observed under basal conditions. This indicates that the initial targeting of cargo may have reached its limit and been compromised due to impaired p62 expression, thereby supporting our hypothesis of defective autophagy induction and degradation machinery.

## 5. Conclusions

The present findings are consistent with previous studies that have linked FA2H activity to several essential cellular processes beyond myelination, such as metabolic pathways associated with autophagy. The consistency of our observations with these studies lends further support to the hypothesis that FA2H plays a role in maintaining cellular integrity through multiple pathways. To elaborate further, our results highlight the complex interplay between FA2H and the autophagy pathway. In particular, the impaired fusion of autophagosomes and lysosomes observed in FA2H-deficient models indicates a broader impact on cellular homeostasis and degradation processes. The altered expression of LC3B and p62 indicates the potential for disruption to the autophagic flux, which could have subsequent effects on cellular health and function. Furthermore, the age-related decline in FA2H expression and its associated effects on myelination and autophagy highlight the progressive nature of FAHN pathology. This temporal aspect of FA2H activity indicates that therapeutic interventions may require not only the correction of the initial deficiencies in FA2H function but also the mitigation of the subsequent impacts on cellular maintenance mechanisms. Further research should concentrate on determining the precise molecular mechanisms responsible for the age-related changes in FA2H expression and their relationship to neurodegeneration. In conclusion, our study provides significant insights into the pathophysiological mechanisms of FAHN, emphasising the critical role of FA2H in myelination and autophagy. The generation of FAHN patient-derived neuronal cells has enabled a detailed examination of these processes, revealing key differences in protein expression, localisation, and cellular interactions. These findings pave the way for future studies aimed at developing targeted therapies to mitigate the effects of *FA2H* mutations and improve outcomes for patients with FAHN.

## Figures and Tables

**Table 1:** List of primary antibodies.

Name	Host	Dilution	Company	Reference No#
Anti-O4-APC FACS	Mouse	1:50	Miltenyi Biotec	130-095-891
BETA III TUBULIN	Mouse IgG	1:100	Santa Cruz	SC51670
FA2H	Rabbit IgG	1:250	Invitrogen	PA5-24728
GAPDH	Mouse IgG	1:10,000	Abcam	AB8245
GFAP	Rabbit IgG	1:500	Agilent	Z0334
KCNQ2/KV7.2	Mouse IgG	1:100	Abcam	AB22897
LC3B (E7X4S)	Rabbit IgG	1:1000	Cell Signaling	43566S
MAP2	Chicken IgY	1:2000	Abcam	AB5392
MBP	Chicken IgY	1:100	ThermoFisher	AB9348
MBP aa 82–87	Rat IgG	1:75	EMD Millipore	MAB386
O4	Mouse IgM	1:100	R&D Systems	MAB1326
PAX6	Rabbit IgG	1:1000	Abcam	AB5790
PLP	Rabbit IgG	1:100	Abcam	AB_9311
SOX2	Rabbit IgG	1:500	Abcam	AB92494
SOX10	Goat IgG	1:100	R&D Systems	AF2864
SQSTML/P62	Rabbit IgG	1:500	Abcam	ABl09012

## Data Availability

The original contributions presented in this study are included in the article. Further inquiries can be directed to the corresponding author.

## Abbreviations

The following abbreviations are used in this manuscript:

BafA1	Bafilomycin A1
BSA	Bovine serum albumin
CNS	Central nervous system
ER	Endoplasmic reticulum
FAHN	Fatty-acid-hydroxylase-associated neurodegeneration
FA2H	Fatty acid 2-hydroxylase
FGF2	Basic fibroblast growth factor
GAPDH	Glyceraldehyde-3-phosphate dehydrogenase
GFAP	Glial fibrillary acid protein
GSL	Glycosphingolipid
GlcCer	Glucosylceramide
hiPSC	Human induced pluripotent stem cell
IF	Immunofluorescence
iPSC	Induced pluripotent stem cell
JXP	Juxtaparanodal junction
KO	Knockout
LC3BI/II	Microtubule-associated proteins 1A/1B light chain 3B
LSM	Laser scanning microscope
MAG	Myelin-associated glycoprotein
MAP2	Microtubule-associated protein 2
MBP	Myelin-binding protein
MOG	Myelin oligodendrocyte glycoprotein
NBIA	Neurodegeneration with brain iron accumulation
NDCs	Neuronal differentiated cells
NIM	Neural induction medium
NPCs	Neural progenitor cells
OLs	Oligodendrocytes
OMIM	Online Mendelian Inheritance in Man
OPCs	Oligodendrocyte progenitor cells
PBS	Phosphate-buffered saline
PDGFRα	Platelet-derived growth factor receptor alpha
PFA	Paraformaldehyde
PLP	poly-L-ornithine
pre-OPC	Pre-oligodendrocyte progenitor
PNS	Peripheral nervous system
PNJ	Paranodal axoglial junction
PSA-NCAM	Polysialylated-neural cell adhesion molecule
RA	Retinoic acid
SHH	Sonic hedgehog
STR	Short tandem repeat
SQSTM1/p62	Sequestosome-1
